# Supplementary material for: Tead1a Initiates Transcriptional Priming Through the TEAD1a/YAP‐Notch1‐Spi1/Cebpα Axis to Promote Neutrophil Fate
Source: Adv Sci (Weinh). 2025 Aug 28;12(41):e05441. doi: 10.1002/advs.202505441 (PMC12591127; doi:10.1002/advs.202505441)
Supplement: Supplementary file 4 — Supplementary Dataset [file ADVS-12-e05441-s004.zip › 3-5.zip!/3-5/02-5.cluster/cluster12/Enrich/ego_CC.pdf]

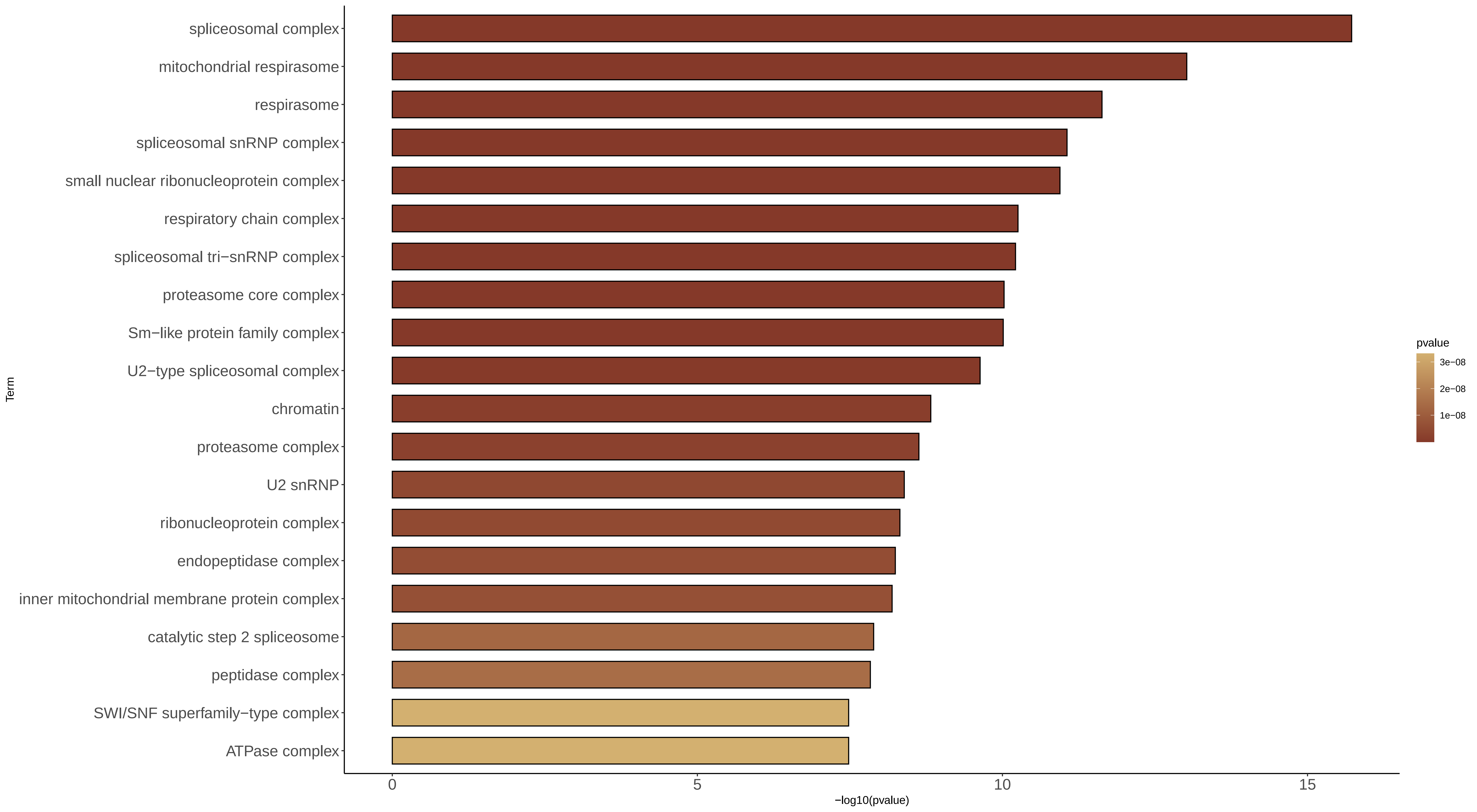
spliceosomal complex
mitochondrial respirasome
respirasome
spliceosomal snRNP complex
small nuclear ribonucleoprotein complex
respiratory chain complex
spliceosomal tri−snRNP complex
proteasome core complex
Sm−like protein family complex
U2−type spliceosomal complex
chromatin
proteasome complex
U2 snRNP
ribonucleoprotein complex
endopeptidase complex
inner mitochondrial membrane protein complex
catalytic step 2 spliceosome
peptidase complex
SWI/SNF superfamily−type complex
ATPase complex
Term
0
5
10
15
−log10(pvalue)
pvalue
3e−08
2e−08
1e−08
